# NbWRKY40 Positively Regulates the Response of *Nicotiana benthamiana* to Tomato Mosaic Virus via Salicylic Acid Signaling

**DOI:** 10.3389/fpls.2020.603518

**Published:** 2021-01-15

**Authors:** Yaoyao Jiang, Weiran Zheng, Jing Li, Peng Liu, Kaili Zhong, Peng Jin, Miaoze Xu, Jian Yang, Jianping Chen

**Affiliations:** ^1^College of Plant Protection, Fujian Agriculture and Forestry University, Fuzhou, China; ^2^State Key Laboratory for Managing Biotic and Chemical Treats to the Quality and Safety of Agro-Products, Institute of Plant Virology, Ningbo University, Ningbo, China; ^3^State Key Laboratory Breeding Base for Zhejiang Sustainable Pest and Disease Control, Zhejiang Provincial Key Laboratory of Plant Virology, Institute of Virology and Biotechnology, Zhejiang Academy of Agricultural Sciences, Hangzhou, China; ^4^College of Plant Protection, Hunan Agricultural University, Changsha, China

**Keywords:** NbWRKY40, salicylic acid, VIGS, tomato mosaic virus, transcription factor

## Abstract

WRKY transcription factors play important roles in plants, including responses to stress; however, our understanding of the function of *WRKY* genes in plant responses to viral infection remains limited. In this study, we investigate the role of *NbWRKY40* in *Nicotiana benthamiana* resistance to tomato mosaic virus (ToMV). *NbWRKY40* is significantly downregulated by ToMV infection, and subcellular localization analysis indicates that NbWRKY40 is targeted to the nucleus. In addition, NbWRKY40 activates W-box-dependent transcription in plants and shows transcriptional activation in yeast cells. Overexpressing *NbWRKY40* (OEWRKY40) inhibits ToMV infection, whereas *NbWRKY40* silencing confers susceptibility. The level of salicylic acid (SA) is significantly higher in OEWRKY40 plants compared with that of wild-type plants. In addition, transcript levels of the SA-biosynthesis gene (*ICS1*) and SA-signaling genes (*PR1b* and *PR2*) are dramatically higher in OEWRKY40 plants than in the control but lower in *NbWRKY40*-silenced plants than in the control. Furthermore, electrophoretic mobility shift assays show that NbWRKY40 can bind the W-box element of *ICS1*. Callose staining reveals that the plasmodesmata is decreased in OEWRKY40 plants but increased in *NbWRKY40*-silenced plants. Exogenous application of SA also reduces viral accumulation in *NbWRKY40*-silenced plants infected with ToMV. RT-qPCR indicates that NbWRKY40 does not affect the replication of ToMV in protoplasts. Collectively, our findings suggest that NbWRKY40 likely regulates anti-ToMV resistance by regulating the expression of SA, resulting in the deposition of callose at the neck of plasmodesmata, which inhibits viral movement.

## Introduction

Plants, as sessile organisms, are often seriously affected by biotic stressors and, thus, have evolved a wide array of sophisticated mechanisms to mitigate the detrimental effects of these stressors, many of which involve transcription factors (TFs) (Miller et al., 2017). Indeed, studies show that TFs are essential components of a plant’s stress response, and they function by binding specific *cis*-acting elements to regulate the expression of genes containing such elements in their promoters (Jakoby et al., 2002; Pandey and Somssich, 2009; Tiwari et al., 2012; Li J. et al., 2019).

The WRKY TFs are plant-specific transcriptional regulators involved in a variety of signaling pathways (Rushton et al., 2010; Song et al., 2016). WRKY TFs that contain WRKY domains and zinc-finger motifs can be categorized into five groups: I, IIa–IIb, IIc, IId–IIe, and III (Liang et al., 2017). All WRKY factors contain a WRKYGQK sequence motif and exhibit a high binding affinity to W-box sequences [TTGAC(C/T)], which are found upstream of many defense-related genes (Rushton et al., 2010) as well in the promoters of WRKY TFs themselves. Therefore, WRKY TFs possess regulatory, auto-regulatory, and cross-regulatory properties (Eulgem et al., 1999). Indeed, since the first *WRKY* gene *SPF1* was identified in sweet potato (Ishiguro and Nakamura, 1994), many WRKY TFs have been reported to be transcriptional regulators that are involved in complex and interconnected transcriptional networks in plants (Rushton et al., 2010).

Tremendous progress has been made in elucidating the function of WRKY proteins associated with abiotic and biotic stress responses. AtWRKY25, AtWRKY26, OsWRKY11, and OsWRKY30 are associated with heat-, salt-, and drought-tolerance (Wu et al., 2009; Li et al., 2011; Shen et al., 2012; Scarpeci et al., 2013). OsWRKY42 is associated with high temperature- and salinity-tolerance (Pillai et al., 2018), and OsWRKY76 functions as a negative regulator in responses to cold stress and blast disease (Yokotani et al., 2013). AtWRKY70 is reported to function at the intersection of the salicylic acid (SA)- and jasmonic acid (JA)-mediated defense-signaling pathways during resistance against specific bacterial and fungal pathogens (Li et al., 2004). In addition, AtWRKY46 is reported to improve basal resistance against *Pseudomonas syringae* through its overlapping and synergetic functions with AtWRKY70 and AtWRKY53 (Hu et al., 2012). Many WRKY proteins are also involved in plant responses to viral infection. For example, *CaWRKYd*, a *Capsicum annuum* gene, is reported to contribute to tobacco mosaic virus (TMV)-mediated hypersensitive-response (HR) apoptosis by regulating downstream gene expression (Huh et al., 2012). However, βC1 proteins encoded by begomoviruses have been shown to bind to WRKY20, interfering with the biosynthesis and accumulation of glucosinolates in plants as well as several defensive factors. This not only benefits the begomovirus, but also whiteflies (i.e., the vector of begomoviruses), and deters non-vector insects (Zhao et al., 2019).

Several *WRKY* genes are reported to participate in various plant hormone-mediated signaling pathways (Ishihama and Yoshioka, 2012; Nan and Gao, 2019). GhWRKY15-overexpressing plants exhibit enhanced resistance to TMV and cucumber mosaic virus (CMV) through the regulation of ROS signaling pathways (Yu et al., 2012). In *Arabidopsis*, AtWRKY8 is reported to mediate crosstalk between ABA and ethylene (ET) signaling pathways by directly regulating the expression of *abscisic acid insensitive 4* (*ABI4*), *1-aminocyclopropane-1-carboxylic acid synthase 6* (*ACS6*), and *ethylene response factor 104* (*ERF104*), thereby enhancing the defense response against TMV-cg (Chen et al., 2013).

Salicylic acid (SA) is a key plant defense hormone that has a remarkable impact on plant defense against various pathogens (Zhang and Li, 2019). Pathogen-induced SA is synthesized either from isochorismate synthase or via the phenylpropanoid biosynthesis pathway (Wildermuth et al., 2001; Ullah et al., 2019). SA was first reported as an inducer of plant disease resistance against TMV in tobacco (White, 1979). Since then, more and more studies show that SA plays an important role as a signaling molecule in plant defense responses against viral infection (Zhou et al., 2014; Yang L. et al., 2016; Li T. et al., 2019; Shaw et al., 2019). SA-mediated defense responses are usually associated with the induction of a number of pathogenesis-related (PR) proteins, which are usually considered to be markers for SA-mediated resistance to viral infection (Whitham et al., 2003; Love et al., 2005). An increasing body of evidence suggests that Non-expressor of PR1 (NPR1), NPR3, and NPR4 serve as SA receptors participating in two opposite signaling pathways downstream of SA (Wu et al., 2012; Ding et al., 2018).

Previous studies also show that WRKY proteins participate in SA-mediated plant immunity. For example, in *Arabidopsis*, 49 of the 74 *WRKY* genes were modulated when plants were treated with SA (Dong et al., 2003). In *Arabidopsis*, *AtWRKY18* is a positive regulator of defense-related gene expression and is reported to increase resistance to the bacterial pathogen *P. syringae* (Chen and Chen, 2002). AtWRKY18, AtWRKY40, and AtWRKY60 are structurally similar and somewhat functionally redundant, and double or triple mutants of these proteins are reported to exhibit reduced resistance to *P. syringae* but greater resistance to *Botrytis cinerea* (Xu et al., 2006). Furthermore, the resistance of *NbWRKY40*-silenced plants to *Phytophthora parasitica* and *B. cinerea* can be induced in *Nicotiana benthamiana* through the SA-mediated signaling pathway (Ma et al., 2016). Interestingly, W-boxes are also found in the promoters of SA-biosynthesis genes and SA-signaling genes, such as *NPR1*, *PR2*, *PR10*, and *Isochorismate synthase 1* (*ICS1*) (Yu et al., 2001; Li et al., 2004; van Verk et al., 2011). However, numerous relationships between WRKY proteins, SA, and viral infections remain largely unclear in plants.

The aim of this study is to analyze the regulation pattern of *NbWRKY40* cloned from *Nicotiana benthamiana* in response to tomato mosaic virus (ToMV) infection and to identify related genes in order to elucidate potential antiviral mechanisms and to determine the involvement of SA-mediated pathways. Overall, the results of our study provide molecular evidence regarding the potential contributions of WRKY TFs to antiviral defense signaling in plants.

## Materials and Methods

### Plant Materials, Growth Conditions, and Treatments

*Nicotiana benthamiana* plants were grown in a greenhouse (26 ± 1°C, 60% RH) under long-day conditions (16 h light/8 h dark). *Agrobacterium* cultures that contained GFP-tagged recombinant ToMV (ToMV-GFP) derivatives were used to infect *N. benthamiana* plants (Liu et al., 2014). Cultures were shaken overnight at 28°C, pelleted, resuspended in an induction buffer (1 M MgCl_2_ and 10 mM MES, pH = 5.6, and 100 mM acetosyringone), diluted to an OD_600_ of 0.6, incubated at room temperature for 3 h, and then used to infiltrate the fourth leaves of 21-day-old plants. At 3 days postinoculation (dpi), plants were photographed under a 100-W handheld long-wave ultraviolet lamp using a digital camera (Canon EOS R, Tokyo, Japan). Some of the 21-day-old seedlings were also sprayed with an SA buffer (500 μM; Sigma-Aldrich, St. Louis, MO, United States). At appropriate times, total RNA was extracted from SA-treated leaves and stored at −80°C for future analysis. The process was repeated at least three times for each treatment.

### *NbWRKY40* Identification and Analysis

The open-reading-frame sequence of *NbWRKY40* (Niben101Scf04944g05002.1) was downloaded from NCBI^1^. Homologs from other species were identified using an NCBI database sequence matching tool (blastn), and sequences with high similarity were downloaded. DNAMAN version 6.0 (Lynnon Biosoft, Quebec, Canada) was used to generate a multiple sequence alignment. MEGA 7.0 was used to perform a phylogenetic analysis. The neighbor-joining method was used to construct a phylogenetic tree with bootstrap values of 1000 (Kumar et al., 2016).

For *NbWRKY40* gene cloning, total RNA was extracted from *N. benthamiana* seedlings using TRIzol reagent (Invitrogen, Carlsbad, CA, United States) and then treated with DNase (Invitrogen, Carlsbad, CA, United States). The full-length *NbWRKY40* sequence was amplified using reverse-transcription PCR (RT-PCR), specific primers (Supplementary Table 1), and super-fidelity DNA polymerase (Phanta Max, Vazyme Biotech Co., Ltd., Nanjing, China). The purified PCR product was then inserted into a plasmid vector pGEM-T easy and transformed into *E. coli* (pGEM-T Easy vector; Promega, Madison, WI, United States), and the full *NbWRKY40* was sequenced from positive clones.

### Quantitative RT-PCR Analysis

RNA was extracted from leaves or protoplasts using TRIzol reagent (Invitrogen, Carlsbad, CA, United States) and then treated with DNase. cDNA was generated by reverse transcription using a First Strand cDNA Synthesis Kit (TOYOBO, Osaka, Japan), diluted 1:20, and used as a template for quantitative real-time PCR (RT-qPCR). RT-qPCR was performed using an AceQ RT-qPCR SYBR Green Master Mix (Vazyme, Nanjing, China) with the *ubiquitin-conjugating enzyme* (*UBC*) as an internal reference gene (Supplementary Table 1). At least three biological replicates, each with three technical replicates, were used for each treatment. Relative expression levels were calculated using the comparative 2^–ΔΔ*Ct*^ method (Willems et al., 2008). The primers used for RT-qPCR are listed in Supplementary Table 1.

### Generation of *N. benthamiana* Transgenic Lines

Specific primers (Supplementary Table 1) were used to obtain the full-length cDNA of *NbWRKY40*. pCV-GFP, which was constructed in our laboratory, was used in this study (Lu et al., 2011). Next, PCR products were double digested with *Bam*HI and *Sac*I and inserted into the *Bam*HI and *Sac*I sites of pCV-His in which the His tag was used to replace the GFP tag of pCV-GFP to create the fusion construct pCV-*NbWRKY40*-His. The recombinant clone was then transferred into *Agrobacterium tumefaciens GV3101* for transformation of *N. benthamiana*. *NbWRKY40* transgenic plants were confirmed by PCR amplification with *NbWRKY40*-specific primers until T2 generation.

### Transcriptional Activation Activity Analysis

Full-length *NbWRKY40* cDNA was generated using primers pGBKT7-NbWRKY40-F and pGBKT7-NbWRKY40-R (Supplementary Table 1), double digested using *Eco*RI and *Bam*HI, and inserted into the *Eco*RI and *Bam*HI sites of pGBKT7. The resulting fusion construct pGBKT7-NbWRKY40 as well as the pGBKT7-*Solanum lycopersicum Abscisic acid insensitive 3*-F (SlABI3-F) vector (positive control) (Gao et al., 2013, 2020) and the empty pGBKT7 vector (negative control) were then transformed into yeast receptor state Y2HGold using the PEG-LiAC method (Yang et al., 2010). The transformants were screened by streaking on SD medium lacking tryptophan (SD/-Trp) and on SD/-Trp medium with X-alpha-gal (SD/-Trp-X-α-Gal) with two replicates of each transformant on each plate. The pCAMBIA1300-35Smini-GUS recombinant plasmid, which was used as a reporter plasmid, was constructed by synthesizing three tandem W-box sequences (5′-CGTTGACCGTTGACCGAGTTGACCTTTTTA- 3′), ligating the fragment to the N-terminus of the CaMV 35S minimal promoter, and then substituting the resulting W-box-35S mini promoter for the CaMV 35S promoter in the pCAMBIA1300 vector. A mutant W-box (mW-box-35S mini, 5′-CGTAGACGGTAGACGGAGTAGACGTTTTTA-3′) was used as a negative control. Meanwhile, the pCB1300-NbWRKY40 recombinant plasmid, which was used as an effector plasmid, was constructed by amplifying the full *NbWRKY40* cDNA, double digesting the product using *Eco*RI and *Bam*HI, and then ligating the fragment to the *Eco*RI and *Bam*HI sites of the pCAMBIA1300 vector. The effector and reporter plasmids were cotransformed into the *A. tumefaciens* strain *GV3101* as described by (Yang et al. (2000), and the resulting strain was used to infiltrate *N. benthamiana* (Yang et al., 2000). At the same time, the effector SlWRKY8 and the reporter plasmids (W–box–35Smini–GUS) were cotransformed into the *A. tumefaciens* strain *GV3101* as a positive control. GUS histochemical analysis was performed as described previously (Chen et al., 2013).

### Subcellular Localization of NbWRKY40

To observe the subcellular localization pattern of NbWRKY40, the full–length NbWRKY40 protein was amplified using specific primers (Supplementary Table 1) and ligated to the N–terminus of GFP in the pCV–GFP to construct the plasmid pCV–NbWRKY40. Then, both pCV–NbWRKY40 and pCV–GFP were transformed individually into *A. tumefaciens* strain *GV3101* by electroporation, and the fourth leaves of 21–day–old *N. benthamiana* plants were infiltrated with dual–transformed *Agrobacterium.* At 2 dpi, infiltrated leaves were collected and evaluated for GFP fluorescence under a Leica TCS SP5 confocal laser scanning microscope (Leica Microsystems, Heidelberg, Germany). Digital images were captured and postacquisition image processing was performed using Adobe Photoshop version 7.0 (Adobe Systems, Inc., San Jose, CA, United States).

### Hormone Treatment

Twenty-one-day-old *N. benthamiana* seedlings were sprayed with either 500 μM SA (Sigma–Aldrich, PCode Number 101998016) diluted in sterile distilled water containing 0.1% Triton X–100 or 0.1% Triton X–100 alone (control). After 12 h, samples were inoculated using ToMV–GFP. At 4 dpi, *N. benthamiana* plants were photographed under long–wavelength UV light using a Canon digital camera, and leaves were harvested for further analysis.

### Hormone Extraction and Analysis

Levels of ABA, SA, and JA extracted from plants were measured and analyzed by Zoonbio Biotechnology Co., Ltd. (Nanjing, China) as described previously (Forcat et al., 2008; Fu et al., 2012). *N. benthamiana* samples of approximately 1 g were ground in a precooled mortar containing 10 mL of an extraction buffer consisting of isopropanol/hydrochloric acid. Extracts were then shaken at 4°C for 30 min. Next, dichloromethane (20 mL) was added, and the samples were shaken again at 4°C for 30 min and centrifuged at 14,000 *g* at 4°C for 3 min. The organic phase was extracted and dried under liquid nitrogen. The pellets were dissolved in 150 mL methanol (0.1% methane acid) and filtered through a 0.22–mm filter membrane. The purified products were analyzed by performing high–performance liquid chromatography–tandem mass spectrometry (HPLC–MS/MS) using the following parameters: injection volume, 2 mL; spray voltage, 4500 V; air curtain pressure, 15 psi; atomizer pressure, 65 psi; auxiliary gas pressure, 70 psi; and atomization temperature, 400°C.

### Virus–Induced Gene Silencing (VIGS)

Approximately 300 bp partial fragments of the *NbWRKY40* coding sequence were RT–PCR–amplified using total RNA from *N. benthamiana* as a template using specific primers (Supplementary Table 1), double–digested using *Bam*HI and *Sma*I, and then inserted into the TRV–RNA2 expression vector. Both the resulting TRV:*NbWRKY40* and TRV–RNA1 vectors were electroporated into *A. tumefaciens GV3101* to knock down *NbWRKY40* expression as described previously (Yang et al., 2018). The empty TRV–RNA2 and TRV–RNA1 (TRV:00) were used to generate a negative control.

### Protein Extraction and Analysis

Western blot assays were performed as described by Yang J. et al. (2016). Briefly, total protein was extracted using a lysis buffer (100 mM Tris–HCl, pH 8.8, 60% SDS, 2% β–mercaptoethanol) and protease inhibitor cocktail tablets (1 tablet per 50 mL buffer; Roche), and extracted proteins were separated using 12.5% SDS–PAGE and transferred to nitrocellulose membranes (Life Technologies), which were blocked for 2 h using TBST buffer (150 mM NaCl, 10 mM Tris–HCl, pH 8.0, 0.05% Tween–20) that contained 10% powdered milk. To detect GFP expression, the membranes were incubated for 4 h at room temperature with anti–GFP polyclonal antibodies (monoclonal antibody; 1:2,000 dilution; Quanshijin, Beijing, China), washed three times with TBST buffer, and then incubated with 1:5000 secondary antibody (Sigma) in TBST. Images were captured using Molecular Image ChemiDoc Touch (Bio–Rad, Hercules, United States), and the GFP signal was quantified using ImageJ.

### Analysis of the *cis*–Regulatory Element of SA–Related Genes

In this study, 2000–bp sequences upstream of the translational start sites of the SA–related genes were considered to be promoter sequences. PlantCARE software^2^ was used to predict the *cis*-regulatory elements based on these promoter sequences.

### Plasmodesmal Callose Staining

*Nicotiana benthamiana* epidermal cells injected with aniline blue dissolved in a sodium phosphate buffer at pH 7.5 were incubated in the dark for 5 min. Then, the injected leaf tissue was dissected, washed with sterile water, and observed under a Leica TCS SP5 confocal laser scanning microscope (Leica Microsystems, Heidelberg, Germany).

### *N. benthamiana* Protoplast Isolation and Transfection

*Nicotiana benthamiana* protoplasts were isolated from 21-day-old seedlings. In short, the young leaves were chopped up and immersed in enzyme solution (0.5 M mannitol, 1.5% cellulose RS (Yakult Honsha, Tokyo, Japan), 0.75% macerozyme R10 (Yakult Honsha), 1 mM CaCl_2_, and 0.1% BSA). The mixture was incubated on a shaking incubator (60 rpm) in the dark at room temperature for 4–5 h and then filtered through Miracloth. The protoplasts were pelleted by centrifugation at 200 *g* for 5 min and then resuspended in an equal volume of W5 solution (154 mM NaCl, 125 mM CaCl_2_, 5 mM KCl and 1.5 mM MES, adjusted to pH 5.7), followed by centrifugation and resuspension in MMG solution (0.4 M mannitol, 15 mM MgCl_2_ and 4.7 mM MES, adjusted to pH 5.7). Plasmid DNA (10 or 20 μg) was added to the protoplast solution and transfected with 40% polyethylene glycol (PEG) solution [40% PEG4000 (Sigma-Aldrich, St. Louis, MO, United States, PCode Number, 102078930), 0.4 M mannitol, and 100 mM Ca(NO_3_)_2_] at room temperature for 20 min. W5 solution was gradually added to dilute the PEG solution and then discarded. The transfected protoplasts were incubated overnight at room temperature and then observed under a confocal microscope.

### Electrophoretic Mobility Shift Assay (EMSA)

For the EMSA, specific promoter fragments of SA-related genes containing the W-box were synthesized as biotin end labels. The unlabeled W-box oligonucleotide served as a competitor. The stabilized streptavidin-horseradish peroxidase conjugate was used for super shift identification. The assay was performed using a LightShift^®^ Chemiluminescent EMSA Kit (Thermo Scientific, Waltham, MA, United States) according to the manufacturer’s instructions.

## Results

### *NbWRKY40* Cloning and Expression Characterization During ToMV Infection

**FIGURE 1:**
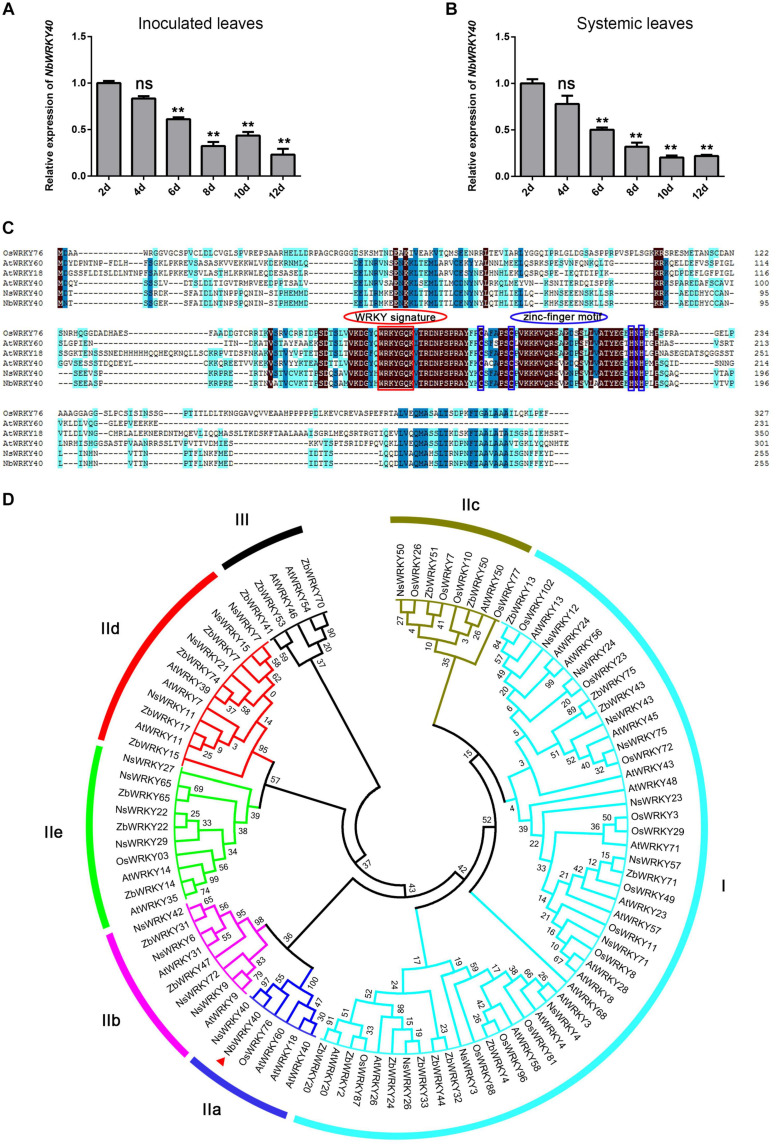
*NbWRKY40* cloning and expression characterization during ToMV infection. **(A)** Time course of *NbWRKY40* levels in inoculated leaves following infection with ToMV. **(B)** Time course of *NbWRKY40* levels in systemic leaves following infection with ToMV. The relative expression of *NbWRKY40* in ToMV-inoculated and systemic leaves was measured using quantitative real-time polymerase chain reaction and the 2^–ΔΔ*Ct*^ method with *Nbubiquitin* as an internal control. Values and error bars represent the mean ± the SD (*n* = 3 with three technical replicates for each biological replicate). **P < 0.01, n.s., not significant, based on Dunnett’s test. **(C)** Sequence alignment of *Oryza sativa* (Os), *Arabidopsis thaliana* (At), *Nicotiana sylvestris* (Ns), and *Nicotiana benthamiana* (Nb) WRKY proteins. Identical amino acids are highlighted in black. **(D)** Phylogenetic tree of *Arabidopsis thaliana* (At), *Nicotiana sylvestris* (Ns), *Nicotiana benthamiana* (Nb), *Oryza sativa* (Os), and *Zanthoxylum bungeanum* (Zb) WRKY proteins. The red triangle indicates NbWRKY40. The phylogenetic tree was constructed using the neighbor-joining method in MEGA 7.0. Numbers at nodes indicate bootstrap values based on 1000 resamplings.

A previous study shows that NbWKRY40 is involved in the *N. benthamiana* response to inoculation with *Phytophthora parasitica* or *Botrytis cinerea* (Ma et al., 2016). In this study, RNA-seq analysis revealed significantly lower levels of *NbWRKY40* expression in *N. benthamiana* after infection with ToMV than in non-infected control plants. Therefore, in order to investigate the effect of ToMV infection on the relative expression of *NbWRKY40* in *N. benthamiana*, we performed RT-qPCR. The results show that the lowest expression levels were detected in inoculated leaves at 12 dpi (0.23-fold) and in systemic leaves at 10 dpi (0.20-fold) compared to controls (Figures 1A,B). These results indicate that *NbWRKY40* expression is suppressed during ToMV infection, potentially to promote ToMV infection.

To further investigate the function of NbWRKY40 in plant resistance, we analyzed the cDNA of *NbWRKY40* (Niben101Scf04944g05002.1) isolated from *N. benthamiana*. RT-PCR confirmed that the cDNA of *NbWRKY40* was 768 bp in length and represented the complete open reading frame. The predicted NbWRKY40 protein was 254 amino acids in length with a molecular weight of 28.95 kDa. Amino acid sequence alignment revealed similarity (37.82–95.29%) to homologs from other species, including *Nicotiana sylvestris* WRKY40 (NsWRKY40, 95.29%), *Arabidopsis thaliana* WRKY60 (AtWRKY60, 46.85%), AtWRKY40 (44.89%), AtWRKY18 (44.74%), and *Oryza sativa* WRKY76 (OsWRKY76, 37.82%) (Figure 1A). Similar to other members of the WRKY group II family, NbWRKY40 also contained a C2H2 motif (C-X5-C-X23-H-X1-H) and a conserved WRKYGQK core sequence (Figure 1C). To demonstrate the evolutionary relationship between NbWRKY40 and WRKY proteins of *O. sativa*, *A. thaliana*, and *N. sylvestris*, a phylogenetic tree was constructed using the neighbor-joining method in MEGA 7.0. The resulting phylogenetic tree indicated that NbWRKY40 was most similar to IIa subgroup proteins, such as AtWRKY60, AtWRKY18, and AtWRKY40 (Figure 1C). Furthermore, we also performed amino acid sequence alignment with homologs NbWRKY40a, NbWRKY40b, NbWRKY40c, NbWRKY40d, and NbWRKY40e, which were analyzed in a previous study (Ma et al., 2016). The sequence alignment showed that the NbWRKY40 cloned in this study shared 100% sequence identity with NbWRKY40e and exhibited 40.87, 29.91, 39.22, and 44.07% sequence identities with NbWRKY40a, NbWRKY40b, NbWRKY40c, and NbWRKY40d, respectively (Supplementary Figure 1).

### NbWRKY40 Activity in Yeast and Plants

**FIGURE 2:**
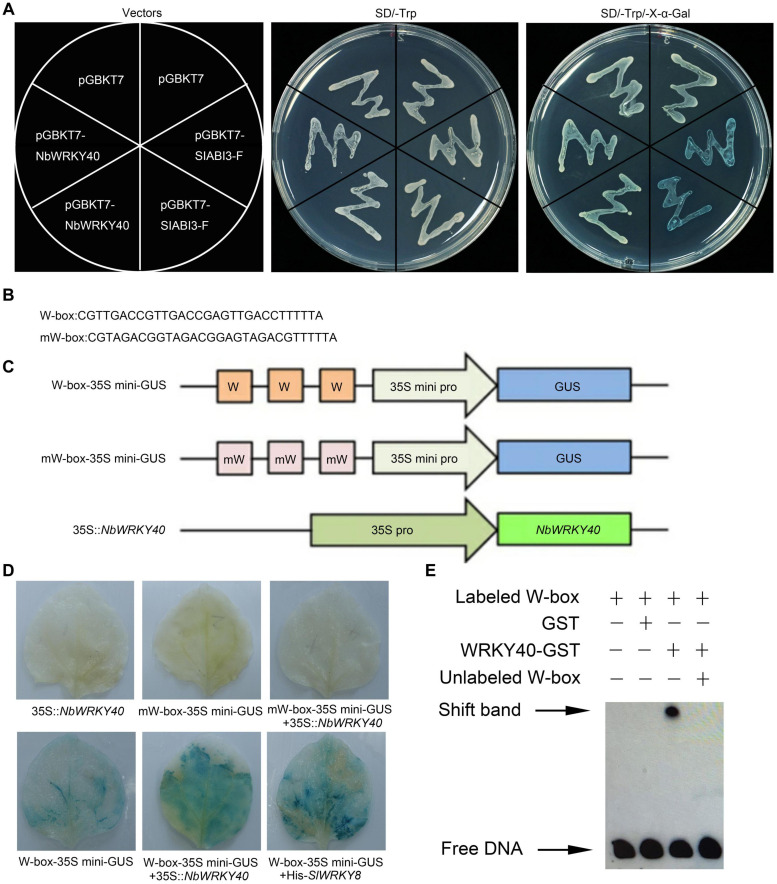
Transcriptional activation ability of NbWRKY40. **(A)** Transcriptional activation ability of NbWRKY40 in yeast cells. **(B)** Triple tandem repeats of the W-box and mW-box. **(C)** Structures of reporter and effector constructs. **(D)** *In vivo* histochemical analysis of GUS activity in cotransfected *Nicotiana benthamiana* leaves. GUS staining was analyzed in the fourth leaves of 28-day-old plants that were *Agrobacterium*-infiltrated with the reporter and effector at an OD_600_ of 0.6. **(E)** Electrophoretic mobility shift assay (EMSA) analysis showing that NbWRKY40 binds to the W-box promoter *in vitro*. The “shift band” black arrow indicates the binding of NbWRKY40 to the biotin-labeled W-box promoter. The “+” indicates the presence of the corresponding component, whereas “–” indicates the absence of the corresponding component.

In order to determine the transcriptional activity of the NbWRKY40 protein, a yeast one-hybrid assay was performed. Yeast cells transformed with pGBKT7-NbWRKY40, the empty pGBKT7 vector (negative control), or the abscisic acid insensitive pGBKT7-SlABI3-F vector (positive control) were all able to form white colonies on the SD/-Trp medium (Figure 2A). However, when transformants were streaked on the SD/-Trp/X-α-Gal medium, only the yeast transformant carrying pGBKT7-SlABI3-F was able to form colonies that turned blue, whereas transformants containing an empty pGBKT7 vector or pGBKT7-NbWRKY40 formed white colonies (Figure 2A). These results suggest that the NbWRKY40 protein did not possess transcriptional activation activity in yeast.

As previously reported, WRKY TFs can bind the *cis*-element W-box to regulate gene transcription activity (Rushton et al., 2010). Based on this hypothesis, a transient co-expression assay was performed to determine whether the transcription of genes containing the *cis*-element W-box in their promoter sequences could be activated by NbWRKY40 in plant cells (Figures 2B,C). Tobacco leaves that were cotransformed with W-box-35Smini-GUS and 35S:*Nb*WRKY40 exhibited strong GUS staining like that observed for leaves that were cotransformed with W-box-35Smini-GUS and 35S:SlWRKY8, which was used as a positive control (Gao et al., 2020), whereas those leaves transformed with only the reporter vector (W-box-35Smini-GUS) exhibited much less staining (Figure 2D). No staining was observed in leaves inoculated with mW-box-35Smini-GUS and/or 35S:NbWRKY40 (Figure 2D). These results indicate that *GUS* transcription can be activated by the binding of NbWRKY40 to W-box motifs.

To further confirm the binding of NbWRKY40 to the W-box element, an EMSA was performed. The analysis indicates that NbWRKY40 was bound to the W-box, but this did not occur in the presence of excess unlabeled W-box (Figure 2E). GST alone was used as a negative control to confirm protein-DNA specificity. Taken together, these results indicate that NbWRKY40 functions as a TF that activates the expression of genes that contain the W-box element in their respective promoter sequences.

### Effect of ToMV on NbWRKY40 Localization

**FIGURE 3:**
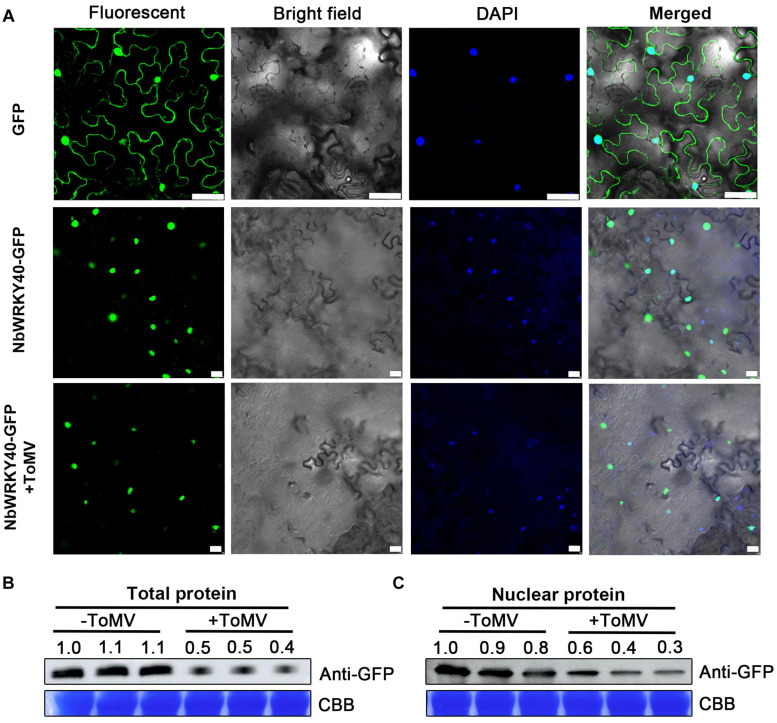
Subcellular localization of NbWRKY40 in *Nicotiana benthamiana* following infection with ToMV. **(A)** Subcellular localization of NbWRKY40 in response to ToMV infection. *Agrobacterium* infiltration was used to introduce recombinant plasmid NbWRKY40-GFP + ToMV into healthy *N. benthamiana* epidermal cells. Healthy leaves infiltrated with an *Agrobacterium* culture containing NbWRKY40-GFP or infiltrated with an *Agrobacterium* culture that only contained GFP acted as controls. The confocal microscopy images were captured 2 dpi under bright-field fluorescence to show cell morphology; under dark field to show green fluorescence, indicating localization of the NbWRKY40 protein, and blue fluorescence, indicating nuclei stained blue by 4,6-diamidino-2-phenyl-indole dihydrochloride (DAPI); and under combination fluorescence to show the three images merged. Scale bar, 100 μm. **(B)** Immunoblot of total protein extracted from the leaves of ToMV-inoculated (+ToMV) and mock-inoculated (–ToMV) plants. Anti-GFP was used to detect GFP fractions. Equal amounts of protein were used for immunoblotting and for staining with Coomassie blue (CBB). **(C)** Immunoblot of nuclear protein extracted from the leaves of ToMV-inoculated and mock-inoculated plants. Anti-GFP was used to detect GFP fractions. Equal amounts of protein were used for immunoblotting and for staining with CBB.

To examine the subcellular localization of NbWRKY40, a fusion plasmid (NbWRKY40-GFP) containing a C-terminal GFP tag was constructed and introduced into *N. benthamiana* epidermal cells using *Agrobacterium* infiltration. At 2 dpi, NbWRKY40-GFP was mainly localized in the nuclei as demonstrated by co-localization with DAPI (Figure 3A). Next, to determine whether the localization of NbWRKY40 could be affected by ToMV infection, NbWRKY40-GFP was co-infiltrated with ToMV into *N. benthamiana* epidermal cells by *Agrobacterium* infiltration. At 2 dpi, strong GFP fluorescence was observed in the nuclei of plants infiltrated with either NbWRKY40-GFP alone or co-infiltration with ToMV (Figure 3A). However, western blot assays revealed that NbWRKY40 expression levels were lower in plants under ToMV stress than those in the mock-inoculated controls (Figures 3B,C).

### Effect of *NbWRKY40* Expression on ToMV Accumulation

**FIGURE 4:**
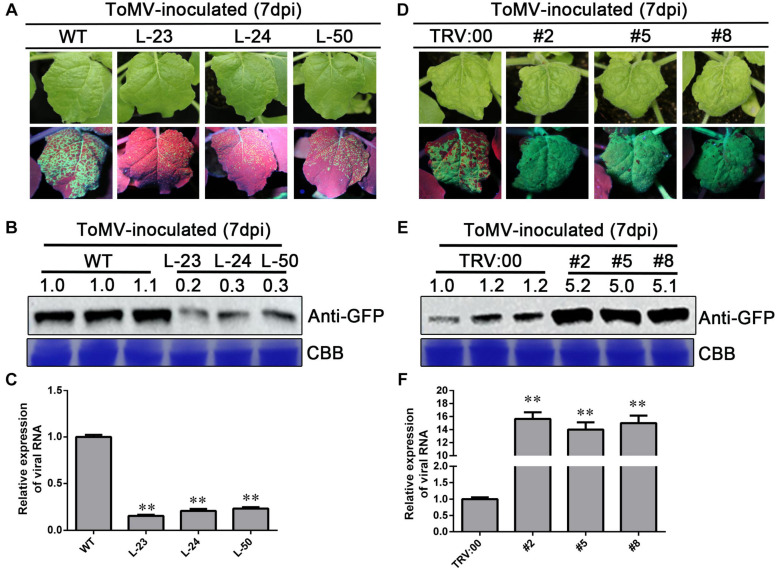
Expression level of *NbWRKY40* affects ToMV accumulation in *Nicotiana benthamiana.* **(A)** Systemic ToMV-GFP fluorescence in wild-type (WT) and T2-homozygous transgenic (L-23, L-24, and L-50) plants at 7 days postinfiltration (dpi). **(B)** Western blot analysis of GFP accumulation in ToMV-GFP-inoculated WT, L-23, L-24, and L-50 plants at 7 dpi. Coomassie blue (CBB)-stained rubisco gel and ImageJ (United States National Institutes of Health, http://rsb.info.nih.gov/nih-image/) were used to determine protein loading. **(C)** Relative expression level of *ToMV-CP* in *NbWRKY40* overexpressing plants. **(D)** Systemic ToMV-GFP fluorescence of TRV:00- and TRV:*WRKY40* (#2, #5, #8)-treated plants at 7 dpi. **(E)** Western blot analysis of GFP expression at 7 dpi. CBB-stained rubisco gel and ImageJ were used to determine protein loading. **(F)** Relative expression level of ToMV-*CP* in *NbWRKY40-*silenced plants. The relative expression level of ToMV-*CP* was calculated using the 2^–ΔΔ*Ct*^ method. The expression level of the *Nbubiquitin* gene in *N. benthamiana* was used as an internal control. Values and error bars represent the mean ± SD of three independent biological replicates with three technical replicates per sample. **P < 0.01 based on Student’s *t*-test.

To further investigate the relationship between *NbWRKY40* expression and ToMV infection, transgenic *N. benthamiana* plants overexpressing *NbWRKY40* lines 23, 24, and 50 (L-23, L-24, and L-50) were constructed and evaluated using RT-qPCR and western blot analysis (Supplementary Figures 2A,B). As expected, higher levels of *NbWRKY40* accumulated in transgenic lines than in WT plants. Next, to determine the role of NbWRKY40 in the plant defense response to ToMV infection, the responses of transgenic and WT plants to ToMV stresses were investigated. L-23, L-24, L-50, and WT plants were inoculated with ToMV-GFP. At 7 dpi, prominent areas of GFP fluorescence were observed in systemic leaves of infiltrated plants. Interestingly, less GFP fluorescence was observed in systemic leaves of L-23, L-24, and L-50 plants infiltrated with ToMV-GFP than in WT plants infiltrated with ToMV-GFP (Figure 4A). Western blot analysis also indicates that lower levels of GFP had accumulated in systemic leaves of L-23, L-24, and L-50 plants inoculated with ToMV-GFP than in the WT (Figure 4B). Transient silencing of *NbWRKY40* was achieved using TRV vector-based VIGS. At 7 dpi, plants infiltrated with TRV1 + TRV2-*NbWRKY40* were confirmed by RT-qPCR (Supplementary Figure 2C). As such, three VIGS (#2, #5, and #8) and vector control (TRV:00) plants were selected for further study (i.e., challenged with ToMV-GFP). At 7 dpi, more GFP fluorescence was observed in systemic leaves of #2, #5, and #8 plants than in those of TRV:00 plants (Figure 4D). Western blot analysis also confirmed that higher levels of GFP had accumulated in the #2, #5, and #8 plants than in plants co-infiltrated with ToMV-GFP and TRV:00 (Figure 4E). Moreover, RT-qPCR analysis also confirmed that the expression level of the ToMV CP was lower in L-23, L-24, and L-50 plants than in WT plants after ToMV infection (Figure 4C), but much higher in #2, #5, and #8 plants (Figure 4F). These findings clearly demonstrate that NbWRKY40 expression levels were negatively related to ToMV accumulation levels in *N. benthamiana.*

### Effect of NbWRKY40 on SA-Related Genes and the Deposition of PD Callose in Plants

**FIGURE 5:**
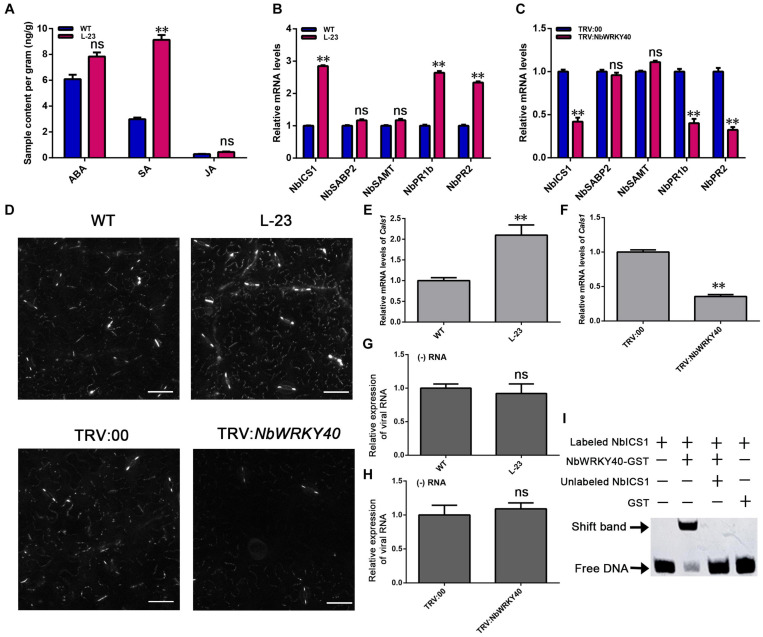
Effect of NbWRKY40 on salicylic acid signaling. **(A)** Hormone levels in the leaves of wild-type (WT) and T2-homozygous transgenic (L-23) *Nicotiana benthamiana*. ABA, abscisic acid; SA, salicylic acid; JA, jasmonic acid. **(B)** Expression of the SA synthesis genes, *ICS1* (isochorismate synthase 1), *SAMT* (SA methyl transferase), and *SABP2* (salicylic acid-binding protein 2), and the SA-dependent signaling-related genes, PR2 and PR1b (pathogenesis-related protein) in WT and L-23 plants. **(C)** Expression of SA-related genes in VIGS and control *N. benthamiana* plants. **(D)** Aniline blue staining of WT-, L-23, TRV:00- and TRV:*NbWRKY40*-treated leaves revealing the callose in plasmodesmata (PDs) and guard cells. **(E)** Transcript levels of the callose biosynthesis gene *callose synthase 1* (*Cals1*) in L-23 plants and WT plants **(E)** and in TRV-*NbWRKY40* plants and TRV:00 plants **(F)**. **(G)** Relative transcript levels of viral RNA in protoplasts of WT and L-23 plants inoculated with ToMV. **(H)** Relative transcript levels of viral RNA in protoplasts of TRV:00 and TRV:*NbWRKY40* plants inoculated with ToMV. **(I)** Binding of WRKY40 to ICS1 promoter fragments. The expression of *Nbubiquitin* was used as an internal control. Values and error bars indicate means ± SD (*n* = 3 with three technical replicates for each biological replicate; **P < 0.01, n.s., not significant, based on Student’s *t*-test).

*WRKY* genes are reported to participate in plant hormone-mediated signaling pathways (Ishihama and Yoshioka, 2012; Nan and Gao, 2019). To assess the effect of NbWRKY40 on plant hormone levels, the ABA, SA, and JA content levels of L-23 and WT plant tissues were compared. L-23 plants contained significantly higher levels of SA than WT plants but similar levels of ABA and JA (Figure 5A). To confirm this finding, RT-qPCR was used to measure the expression levels of SA-related genes. The expression levels of *ICS1*, *PR1b*, and *PR2* were significantly higher in L-23 plants than in WT plants (Figure 5B) but were significantly lower in *NbWRKY40*-silenced plants than in WT plants (Figure 5C).

Callose deposition at plasmodesmata (PD) is regulated by SA (Wang et al., 2013; Cui and Lee, 2016). We used aniline blue staining to assess the amount of callose deposited at PDs. A greater amount of callose was deposited on PDs in L-23 plant leaves than on PDs in WT plant leaves; however, only a very small amount of callose was deposited on PDs in leaves of *NbWRKY40*-silenced plants compared with that in TRV:00-treated, control plants (Figure 5D and Supplementary Figure 3). To understand this phenomenon, we used RT-qPCR to analyze the relative expression of the callose biosynthesis gene *callose synthase 1* (*Cals1*). The transcript level of *Cals1* in L-23 plants was significantly higher than that in WT plants, whereas the expression of *CalS1* was significantly lower in TRV-*NbWRKY40* plants than in TRV:00 plants (Figures 5E,F).

To confirm the possible role of NbWRKY40 in ToMV replication, we conducted protoplast transfection assays. Protoplasts were isolated from *N. benthamiana* and then transfected with ToMV-GFP. At 48 h post transfection, GFP fluorescence was observed using confocal microscopy (Supplementary Figure 4). RT-qPCR was performed to monitor viral (−) RNA accumulation in protoplasts infiltrated with ToMV-GFP. The relative expression levels of viral RNA and GFP fluorescence in WT, L-23, TRV:00, and TRV-*NbWRKY40* plants indicates that NbWRKY40 did not affect the replication of ToMV in protoplasts (Figures 5G,H).

WRKY protein is generally thought to bind to the consensus W-box sequence TTGAC (C/T). In order to identify whether SA-related genes contain the *cis*-regulatory element W-box, the PlantCARE tool was used to analyze the 2000-bp sequence upstream of the putative translation start site of the SA-related genes. As shown in Supplementary Figure 5, the SA-related gene *NbICS1* contains the WRKY identification area of the W-box, which is located at 1673 to 1678 bp upstream of the ATG start codon. As a first step toward the characterization of WRKY40 binding sites in the *NbICS1* gene promoter, we prepared 84-bp promoter fragments that contained a TTGAC (C/T) core sequence in the center. After biotin labeling, the fragments were assayed for their ability to bind NbWRKY40 through EMSA. The results show that NbWRKY40 protein can recognize and bind to the biotin-labeled probes of *NbICS1* to cause mobility changes (Figure 5I). These findings indicate that the NbWRKY40 protein can bind to the W-box in the promoter region of *NbICS1*.

### Effect of SA on ToMV Infection

**FIGURE 6:**
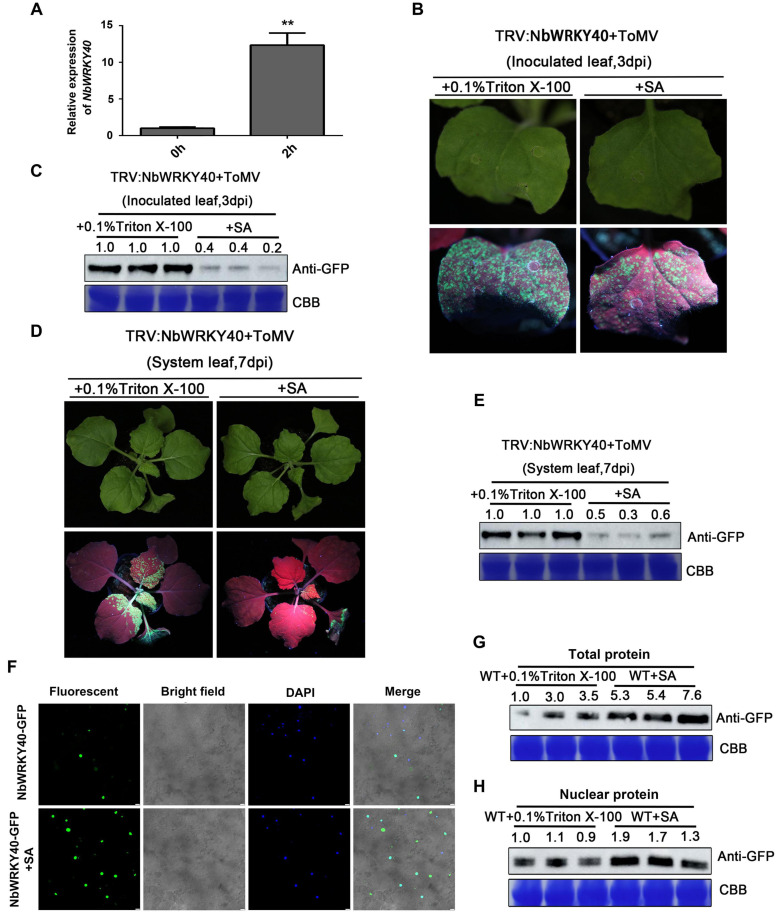
Effect of exogenous salicylic acid (SA) application on viral infection and subcellular localization of NbWRKY40. **(A)** Relative expression of *NbWRKY40* in response to SA treatment **(B)** GFP fluorescence of *NbWRKY40*-silenced leaves inoculated with ToMV-GFP at 3 days postinfiltration (dpi) following SA treatment is less intense than that following 0.1% Triton X-100 treatment alone. **(C)** Western blot analysis of GFP accumulation in ToMV-GFP-inoculated leaves at 3 dpi. Coomassie brilliant blue (CBB)-stained rubisco gel and ImageJ (United States National Institutes of Health, http://rsb.info.nih.gov/nih-image/) were used to determine protein loadings. **(D)** GFP fluorescence of systemic leaves of *NbWRKY40*-silenced plants inoculated with ToMV-GFP at 7 dpi following SA treatment is less intense than that following 0.1% Triton X-100 treatment alone. **(E)** Western blot analysis of GFP accumulation in non-infected leaves of ToMV-GFP-inoculated plants at 7 dpi. CBB-stained rubisco gel and ImageJ (United States National Institutes of Health, http://rsb.info.nih.gov/nih-image/) were used to determine protein loadings. **(F)** Subcellular localization of NbWRKY40 protein in response to SA treatment. The recombinant plasmid *NbWRKY40*-GFP was introduced into *Nicotiana benthamiana* epidermal cells using *Agrobacterium* infiltration. Leaves infiltrated with an *Agrobacterium* culture that only contained GFP acted as controls. The confocal microscopy images were captured under bright-field fluorescence to show cell morphology; under dark field to show green fluorescence, indicating localization of the NbWRKY40 protein, and blue fluorescence, indicating nuclei stained blue by 4,6-diamidino-2-phenyl-indole dihydrochloride (DAPI); and under combination fluorescence to show the three images merged. Scale bar, 100 μm. **(G)** Immunoblot of total protein extracted from the leaves of SA- and mock-treated plants. Anti-GFP was used to detect GFP fractions. Equal amounts of protein were used for immunoblotting and for staining with CBB. **(H)** Immunoblot of nuclear protein extracted from the leaves of SA- and mock-treated plants. Anti-GFP was used to detect GFP fractions. Equal amounts of protein were used for immunoblotting and for staining with CBB. **P < 0.01.

Salicylic acid treatment affects the expression of a large number of *WRKY* genes in *Arabidopsis*, suggesting an important role for WRKY-mediated transcriptional control in gene expression (Dong et al., 2003). We used RT-qPCR to investigate the effect of SA treatment on *NbWRKY40* expression. At 2 h after SA treatment, the expression level of *NbWRKY40* was 12.3-fold higher than that in the control plants (Figure 6A). In addition, to investigate whether an exogenous application of SA can reduce viral accumulation in *NbWRKY40*-silenced plants infected with ToMV, *NbWRKY40*-silenced seedlings were treated with 500 μM SA or 0.1% triton X-100 (which acted as a negative control) before inoculation with ToMV-GFP. At 3 dpi, stronger GFP fluorescence was observed in the inoculated leaves of plants sprayed with 0.1% Triton X-100 than in those sprayed with SA (Figure 6B), and western blot analysis indicated that lower levels of GFP had accumulated in SA-treated leaves than in controls (Figure 6C). At 7 dpi, SA-treated plants exhibited weaker GFP fluorescence and lower levels of GFP accumulation than control plants (Figures 6D,E), which indicates that SA pretreatment can reduce the susceptibility of *NbWRKY40*-silenced plants to ToMV. Moreover, to determine whether the localization of NbWRKY40 could be affected by SA treatment, *N. benthamiana* was pretreated with SA and then infiltrated with NbWRKY40-GFP. At 2 dpi, the NbWRKY40-GFP protein mainly accumulated in the nucleus with stronger GFP fluorescence observed in SA-treated leaves than in control leaves (Figure 6F). Western blot assays also indicated that the protein expression level of NbWRKY40-GFP was higher in plants pretreated with SA than in those that did not receive the SA treatment (Figures 6G,H).

## Discussion

WRKY TFs comprise one of the largest families of plant transcription factors, and many *WRKY* genes have been reported to play important roles in host defense mechanisms (Huh et al., 2012; Ishihama and Yoshioka, 2012). However, the functional roles of WRKYs and their involvement in defense mechanisms against pathogen infection remain unclear. In this study, we cloned a *WRKY* TF gene, *NbWRKY40*, from *N. benthamiana*. Multiple sequence alignments indicated that NbWRKY40 was similar to five other WRKY proteins (OsWRKY76, AtWRKY60, AtWRKY40, AtWRKY18, and NsWRKY40; Figure 1C), and phylogenetic analysis indicated that NbWRKY40 was most similar to subgroup IIa WRKY proteins (e.g., AtWRKY40, AtWRKY18, AtWRKY60, and OsWRKY76; Figure 1D). Among the identified homologs, OsWRKY76 is a negative regulator of defense-related metabolite biosynthesis and affects SA production through its participation in the phenylpropanoid pathway (Liang et al., 2017), which suggests that NbWRKY40 may also be involved in plant defense mechanisms.

Consistent with the domain of WRKY proteins, NbWRKY40 contained both the conserved WRKY motif and a zinc-finger motif in the central region of the genome (Figure 1C). Our confocal microscopy observations indicate that NbWRKY40 targets the nucleus exclusively, at least in the leaf epidermal cells of *N. benthamiana* (Figure 3A). To determine whether NbWRKY40 is a transcriptional activator or repressor, we also performed *Agrobacterium*-mediated transient co-expression analyses, which reveal that NbWRKY40 can activate *GUS* transcription (Figures 2B–D). Furthermore, the binding of NbWRKY40 to the W-box motif is confirmed by EMSA (Figure 2E). Taken together, these results suggest that NbWRKY40 functions as a transcription activator by specifically binding the W-box motif, thereby regulating gene expression.

Previous studies show that the transcripts of *WRKY* genes can be strongly induced by several different pathogens, including *Xanthomonas oryzae*, *Ralstonia solanacearum*, *Coniothyrium diplodiella*, and CMV (Shi et al., 2014; Hwang et al., 2016; Zhang et al., 2016; Zou et al., 2019). However, *NbWRKY40* expression was significantly reduced in ToMV-infected plants (Figures 1A,B). Generally, viral infection also affects the subcellular distribution of the host protein, leading to the loss of normal functions. For example, rice stripe virus (RSV) infection affects the distribution pattern of OsHSP20 in rice cells (Li et al., 2015). Here, we demonstrate that ToMV infection did not affect the localization of NbWYKY40 in the nucleus but did inhibit expression levels of NbWYKY40 in the plant cells (Figure 3A). Furthermore, the constitutive expression of *NbWRKY40* in transgenic plants enhanced the resistance of plants to ToMV infection, whereas *NbWRKY40*-silenced plants were more susceptible to infection (Figure 4). Similarly, many WRKY TFs have been confirmed as positive regulators in responses to viral infection, such as SlWRKY8, AtWRKY30, and CaWRKYd (Dang et al., 2014; Zou et al., 2019; Gao et al., 2020). Taken together, our findings suggest that NbWRKY40 plays a positive role in regulating the defense response of *N. benthamiana* against ToMV infection.

WRKY TFs are known to be associated with the SA-, JA-, and ABA-mediated signaling pathways (Dong et al., 2003). In our study, the SA level was dramatically higher in *NbWRKY40* transgenic plants than in WT (Figure 5A). Pathogen-induced SA is predominantly biosynthesized from chorismate by *ICS1*, followed by the induction of *PR* gene expression (Wildermuth et al., 2001; Whitham et al., 2003; Love et al., 2005; Hao et al., 2018). Our RT-qPCR analyses show that expression levels of *ICS1*, *PR2*, and *PR1b* were significantly more upregulated in the L-23 plants than in WT plants, whereas these genes were downregulated when *NbWRKY40* was silenced (Figures 5B,C). Previous studies report that SA can enhance plant antiviral defense through inhibiting the movement and replication of several plant viruses (Murphy and Carr, 2002; Wong et al., 2002). In this study, we observed that both locally and systemically infected leaves of *NbWRKY40*-silenced plants pretreated with SA were less susceptible to ToMV infection (Figure 6), which suggests that SA is negatively related to ToMV accumulation in *N. benthamiana*. Furthermore, RT-qPCR analyses show that NbWRKY40 did not affect the replication of ToMV in protoplasts, indicating that NbWRKY40 affects viral infection, possibly through inhibiting the movement of ToMV. The movement of plant viruses between cells is controlled by the deposition of callose at the neck of PD during virus infection (Li et al., 2012; Cui et al., 2018). In *Arabidopsis*, SA has been shown to participate in the regulation of callose deposition at PDs by affecting the expression of *Cals1* and *Cals8* (Wang et al., 2013; Cui and Lee, 2016). Here, we find that overexpression of *NbWRKY40* did increase SA levels (Figure 5A). Furthermore, PD callose deposition was significantly higher in L-23 plants than in WT plants; however, levels were significantly lower in *NbWRKY40*-silenced plants than in WT plants (Figure 5F). These results indicate that PD callose deposition levels in *N. benthamiana* plants were related to the accumulation of SA controlled by NbWRKY40. WRKY TFs can regulate gene expression by binding the W-box element at the promoter of genes. WRKY28 and WRKY46 bind to the W-box at the promoters of *ICS1*, leading to increased *ICS1* expression in *Arabidopsis* protoplasts (van Verk et al., 2011). Consistent with these previous findings, our results also indicate that NbWRKY40 functions as an activator through binding the W-box element of *ICS1* promoters (Figures 2, 5) to promote SA synthesis. In addition, the relative expression of *NbWRKY40* transcripts was induced by SA treatment (Figure 6A). The inoculation of *Capsicum annum* with *R. solanacearum* has been reported to induce CaCDPK15 and indirectly activate downstream CaWRKY40, which, in turn, potentiates CaCDPK15 expression, creating a positive feedback loop. It is thought that this positive feedback loop amplifies defense signaling against *R. solanacearum* infections and efficiently activates strong plant immunity (Shen et al., 2016). Thus, we speculate that NbWRKY40 plays a role in a positive feedback loop for SA synthesis to amplify defense signaling. Overall our findings suggest that the NbWRKY40 restricts ToMV infection, possibly through regulating the expression of SA, resulting in the deposition of callose at the neck of PD to inhibit viral movement.

## Conclusion

In this study, we identify a WRKY transcription factor in *N. benthamiana*, namely *NbWRKY40*, that is induced by ToMV infection. *NbWRKY40* overexpression improved resistance to ToMV stress, whereas knockdown of the gene enhanced the susceptibility of NbWRKY-silenced plants to ToMV infection. Analysis of molecular mechanisms involved in enhanced resistance to ToMV revealed the extensive roles of *NbWRKY40* in upregulating the expression of SA-synthesis and/or -responsive genes. Callose staining reveals that PD of overexpression WRKY40 plants were less permeable than those of WT plants, whereas those of NbWRKY40-silenced plants were more permeable than WT. Furthermore, our analyses show that NbWRKY40 did not affect the replication of ToMV in protoplasts. Taken together, our findings suggest that NbWRKY40 may mediate resistance to ToMV, possibly through regulating the expression of SA, resulting in the deposition of callose at the neck of PD to inhibit viral movement.

## Data Availability Statement

The original contributions presented in the study are included in the article/Supplementary Material, further inquiries can be directed to the corresponding author/s.

## Author Contributions

JY and JC conceived the project and designed the experiments. YJ, WZ, and JY conducted the experiments with assistance from JL, PL, KZ, PJ, and MX. All authors analyzed and discussed the results. YJ, JY, and JC wrote the manuscript.

## Conflict of Interest

The authors declare that the research was conducted in the absence of any commercial or financial relationships that could be construed as a potential conflict of interest.

## Footnotes

1 https://www.ncbi.nlm.nih.gov/

2 http://bioinformatics.psb.ugent.be/webtools/plantcare/html/

## Supplementary Material

Supplementary Figure 1 Alignment of the amino acid sequences of the NbWRKY40 protein with the NbWRKY40a (NbS00048676g0011.1), NbWRKY40b (NbS00010134g0012.1), NbWRKY40c (NbS00051158g0004.1), NbWRKY40d (NbS00001817g0013.1), and NbWRKY40e (NbS00036148g0004.1).

Supplementary Figure 2 Assessment of transgene and silencing efficiency in *Nicotiana benthamiana*. **(A)** Western blot analysis of transgenic lines (L-23, L-24, and L-50). **(B)** Relative expression levels of *NbWRKY40* in transgenic lines (L-23, L-24, and L-50) and wild-type (WT) plants. **(C)** Relative expression levels of *NbWRKY40* in VIGS and control *N. benthamiana* plants determined by RT-qPCR.

Supplementary Figure 3 Relative intensity of aniline blue staining of WT-, L- 23-, TRV: 00-, and TRV:NbWRKY40-treated leaves to assess the amount of callose deposited at PDs and guard cells. Error bars represent the SD of the means of three biological repeats. A two-sample unequal variance directional *t*-test was used to test the significance of the difference (^∗∗^*P*-value < 0.01).

Supplementary Figure 4 Confocal microscopy images of GFP fluorescence in *Nicotiana benthamiana* protoplasts 48 h post transfection with ToMV-GFP. Protoplasts were isolated from the transgenic *NbWRKY40* overexpression line 23 (L-23), wild type (WT), transiently *NbWRKY40* silenced plants (TRV:NbWRKY40), and *N. benthamiana* inoculated with TRV:00. Scale bar, 100 μm.

Supplementary Figure 5 Schematic of the W-box element of SA-related gene promoters. Promoter sequences (2000 bp) upstream of genes were chosen for *cis*-regulatory element analysis using the PlantCARE online tool (http://www.dna.affrc.go.jp/PLACE/).

Supplementary Table 1 Primers used in this study.
