# Supplementary figures and images for: NbWRKY40 Positively Regulates the Response of Nicotiana benthamiana to Tomato Mosaic Virus via Salicylic Acid Signaling
Source: Front Plant Sci. 2021 Jan 15;11:603518. doi: 10.3389/fpls.2020.603518 (PMC7857026; doi:10.3389/fpls.2020.603518)

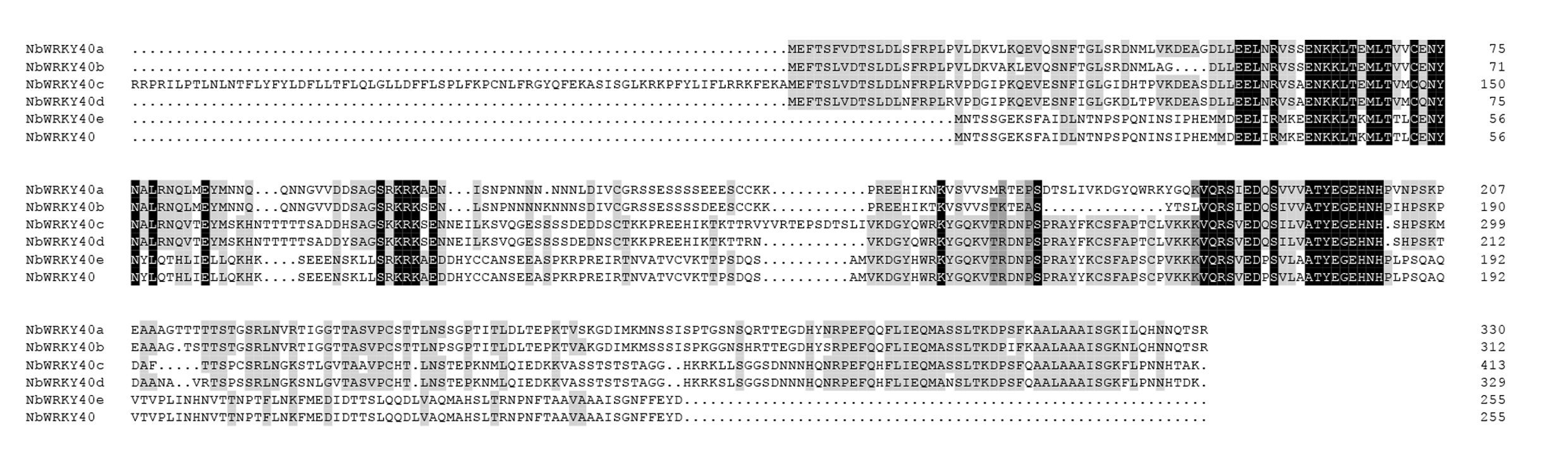

Supplement: Supplementary Figure 1 — Alignment of the amino acid sequences of the NbWRKY40 protein with the NbWRKY40a (NbS00048676g0011.1), NbWRKY40b (NbS00010134g0012.1), NbWRKY40c (NbS00051158g0004.1), NbWRKY40d (NbS00001817g0013.1), and NbWRKY40e (NbS00036148g0004.1). [file Image_1.JPEG]

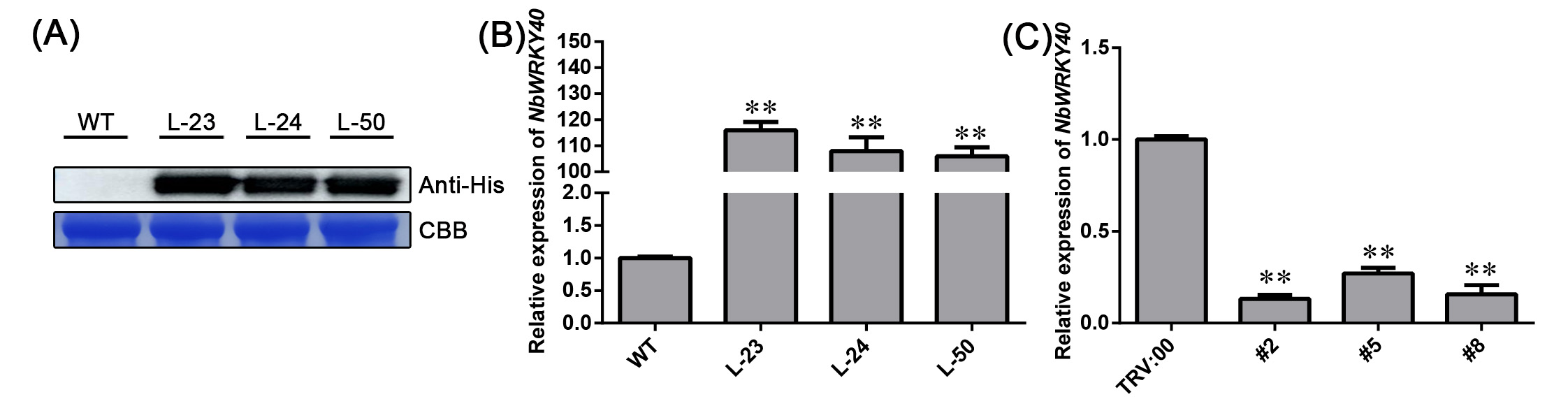

Supplement: Supplementary Figure 2 — Assessment of transgene and silencing efficiency in Nicotiana benthamiana. (A) Western blot analysis of transgenic lines (L-23, L-24, and L-50). (B) Relative expression levels of NbWRKY40 in transgenic lines (L-23, L-24, and L-50) and wild-type (WT) plants. (C) Relative expression levels of NbWRKY40 in VIGS and control N. benthamiana plants determined by RT-qPCR. [file Image_2.JPEG]

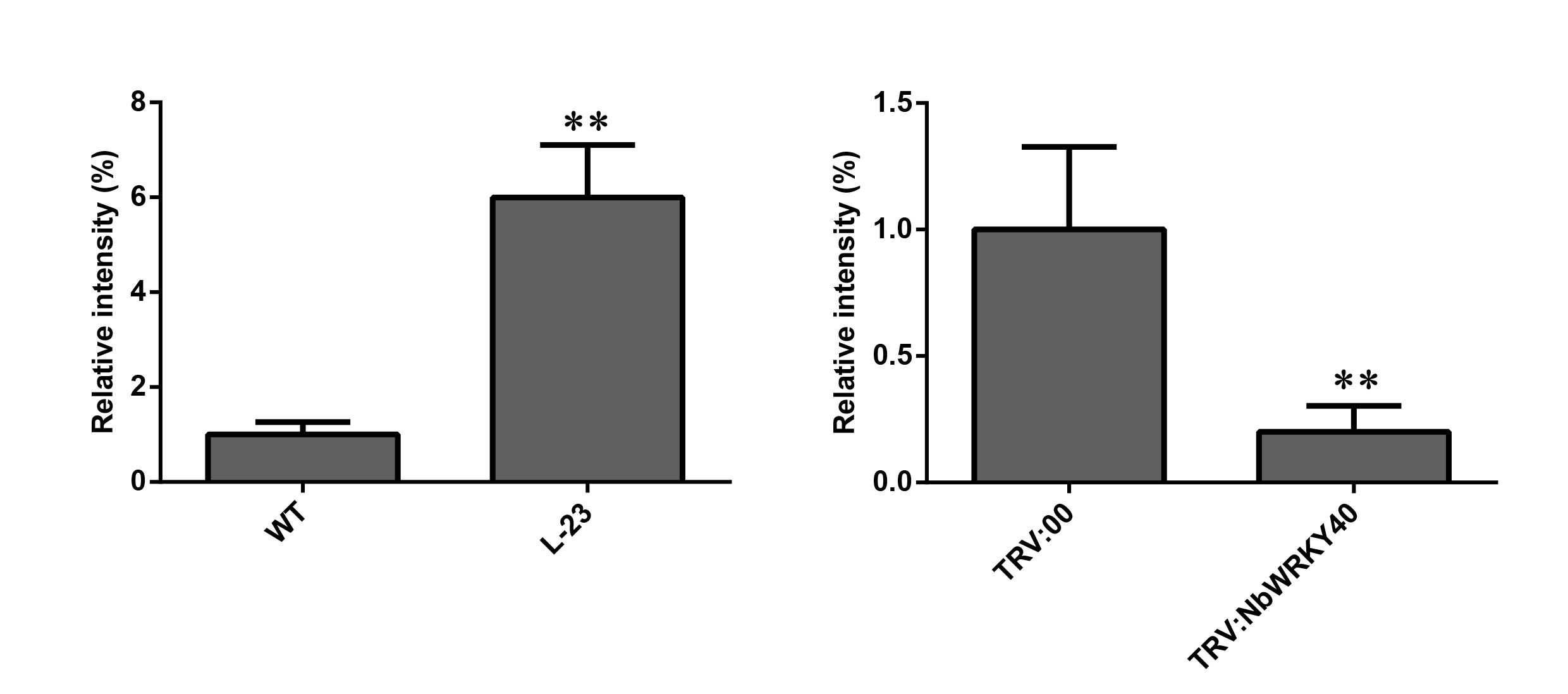

Supplement: Supplementary Figure 3 — Relative intensity of aniline blue staining of WT-, L- 23-, TRV: 00-, and TRV:NbWRKY40-treated leaves to assess the amount of callose deposited at PDs and guard cells. Error bars represent the SD of the means of three biological repeats. A two-sample unequal variance directional t-test was used to test the significance of the difference (∗∗P-value < 0.01). [file Image_3.JPEG]

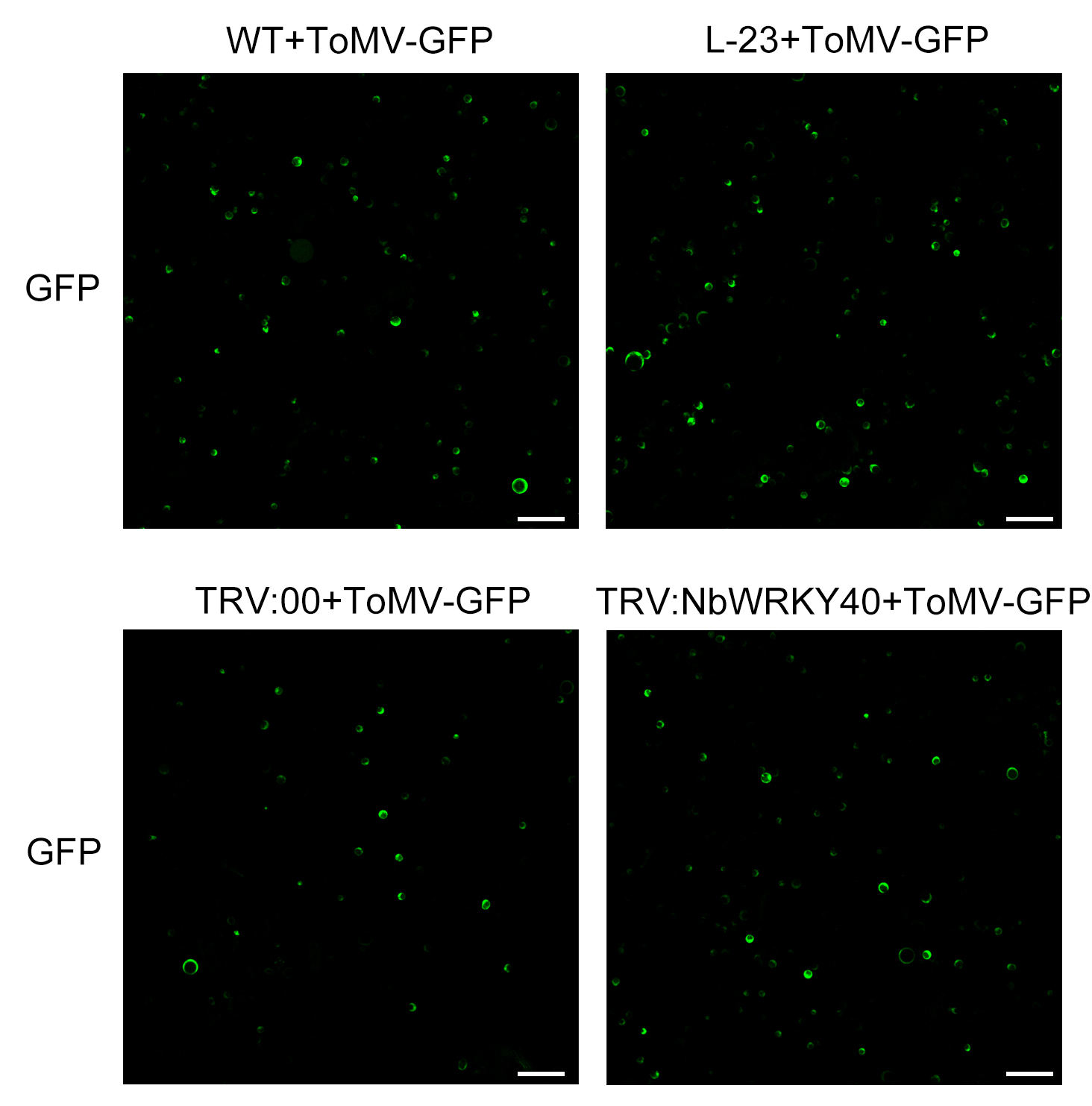

Supplement: Supplementary Figure 4 — Confocal microscopy images of GFP fluorescence in Nicotiana benthamiana protoplasts 48 h post transfection with ToMV-GFP. Protoplasts were isolated from the transgenic NbWRKY40 overexpression line 23 (L-23), wild type (WT), transiently NbWRKY40 silenced plants (TRV:NbWRKY40), and N. benthamiana inoculated with TRV:00. Scale bar, 100 μm. [file Image_4.JPEG]

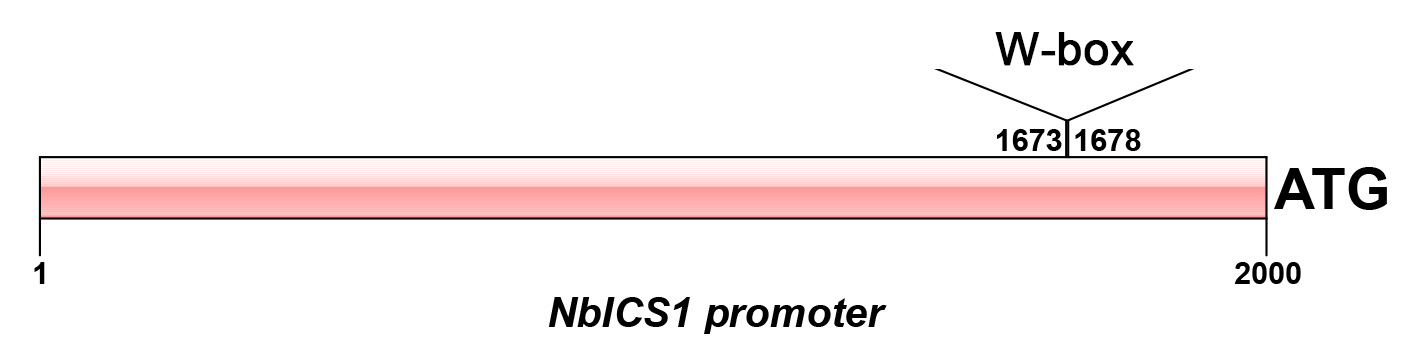

Supplement: Supplementary Figure 5 — Schematic of the W-box element of SA-related gene promoters. Promoter sequences (2000 bp) upstream of genes were chosen for cis-regulatory element analysis using the PlantCARE online tool (http://www.dna.affrc.go.jp/PLACE/). [file Image_5.JPEG]
