# Supplementary material for: NbWRKY40 Positively Regulates the Response of Nicotiana benthamiana to Tomato Mosaic Virus via Salicylic Acid Signaling
Source: Front Plant Sci. 2021 Jan 15;11:603518. doi: 10.3389/fpls.2020.603518 (PMC7857026; doi:10.3389/fpls.2020.603518)
Supplement: Supplementary Table 1 — Primers used in this study. [file Table_1.DOCX]

**Table S1**. Primers used in this study.

| Name | Primer seguence 5'-3' | Use |
| --- | --- | --- |
| TRV-WRKY40F | CGCGGATCCCTGTTGTGCTAATTCAGAAG | VIGS |
| TRV-WRKY40R | TCCCCCGGGCACTGTTACTTGAGCTTG | VIGS |
| pGBKT7-NbWRKY40-F | CCGGAATTCATGAATACAAGTTCTGGGGA | Y2H |
| pGBKT7-NbWRKY40-R | CGCGGATCCTTAATCATATTCAAAAAAATTC | Y2H |
| NbWRKY40-GFP-F | GCGGATCCATGAATACAAGTTCTGGGGA | Subcellular localization |
| NbWRKY40-GFP-R | ACGCGTCGACATCATATTCAAAAAAATTCC | Subcellular localization |
| NbUBC-FRTF | TGGAGGTACATTTAAGCTGACAC | RT-qPCR |
| NbUBC-RTR | TCACAGAGCAAAGACTGGATTG | RT-qPCR |
| NbWRKY40-RTF | TCCGGGAATTTTTTTGAATATGA | RT-qPCR |
| NbWRKY40-RTR | CAGTGAAAAAACAAAACCGTTTGA | RT-qPCR |
| NbICS-RTF | TCATCACTCGTGAAATGGTCG | RT-qPCR |
| NbICS-RTR | GAGGCTGGGAGTTAACCAAGT | RT-qPCR |
| NbSABP2-RTF | ATGCCATGGAGGTTGGAGTTG | RT-qPCR |
| NbSABP2-RTR | ATTCATACCACCAAGACTATG | RT-qPCR |
| NbSAMT-RTF | ATGAATGGAGGAATTGGAGAC | RT-qPCR |
| NbSAMT-RTR | ATGAGCTCTGATACCACTATG | RT-qPCR |
| NbPR2-RTF | GCAGCAGACGATGTAATGATGG | RT-qPCR |
| NbPR2-RTR | TCCACAAGCCTAGTGAGCCTC | RT-qPCR |
| NbPR1b-RTF | GTGGACACTATACTCAGGTG | RT-qPCR |
| NbPR1b-RTR | TCCAACTTGGAATCAAAGGG | RT-qPCR |
| Cals1-RTF | TTGCATTCTTGCTTTCATGC | RT-qPCR |
| Cals1-RTR | GGAACTCCGAAACAAATGGA | RT-qPCR |
| NbICS1promoter | TAGTACAGAATATGAAGAAAGCTTTGCATATGGTGTTTATTGACCTAGAGAAAGTGTACATCAAGGTCCCTAGGGATGTCCTTT | EMSA |
